# Characterization of SiC Ceramic Joints Brazed Using Au–Ni–Pd–Ti High-Temperature Filler Alloy

**DOI:** 10.3390/ma12060931

**Published:** 2019-03-20

**Authors:** Huamin He, Chuanyang Lu, Yanming He, Wenjian Zheng, Jianguo Yang, Limei Wang, Yuan Sun, Zengliang Gao

**Affiliations:** 1 Institute of Process Equipment and Control Engineering, Zhejiang University of Technology, Hangzhou 310014, China; 2111602136@zjut.edu.cn (H.H.); lvcykk@163.com (C.L.); zwj0322@zjut.edu.cn (W.Z.); yangjg@zjut.edu.cn (J.Y.); lmwang@zjut.edu.cn (L.W.); zlgao@zjut.edu.cn (Z.G.); 2 Department of Superalloy, Institute of Metal Research, Chinese Academy of Science, Shenyang 110016, China

**Keywords:** SiC ceramic, high-temperature brazing, microstructure, residual stress

## Abstract

In this work, (Au_79_Ni_17_Pd_4_)_96_Ti_4_ (wt.%) filler alloy was designed and employed to join SiC ceramics. The effects of brazing temperature and soaking time on the microstructure and fracture morphology of joints were investigated. The results show that the joint obtained can be described as SiC/reaction layer/braze/reaction layer/SiC. The reaction layer was composed of TiC and Au (Si, Ti). The wettability of the filler alloy toward the SiC ceramics was analyzed. The braze zone was mainly constituted by Pd_2_Si, Ni_2_Si, and Au (Ni, Si). A large number of nano-sized TiC particles were distributed within the Au (Ni, Si) layer. The formation mechanism of the braze containing different phases was discussed. The brazing temperature and soaking time had a significant effect on the reaction layer at the SiC/braze interface and TiC particles within the Au (Ni, Si) layer, while they showed a negligible effect on the Pd_2_Si and Ni_2_Si within the braze. The inherent reason was also clarified in detail. The joint fractography indicated that a good bonding was achieved between the filler alloy and SiC, while joint fracture was primarily induced by the thermal stresses residing after the brazing cycle.

## 1. Introduction

Developing advanced energies to reduce the consumption of traditional fossil fuels is becoming necessary over the world. Nuclear energy, which is economic and environmentally friendly, was vigorously investigated over the past decades. However, it is still urgent to ensure nuclear safety in consideration of catastrophic accidents [1]. The fuel element, which is the core component in a nuclear reactor, consists of the inner nuclear fuel and outer cladding. The cladding is designed to prevent the nuclear fuel from escaping and being in contact with the coolants. In other words, the cladding is definitely critical to the safety of nuclear reactors. Currently, zirconium alloys are widely adopted as nuclear-fuel cladding materials in commercial light-water reactors (LWR) [2,3]. Unfortunately, they experience metal–water reactions, as revealed in the Fukushima nuclear disaster in 2011. Coupled with the commercialization of the fourth generation of nuclear energy, much attention is focused on the development of advanced materials for nuclear-fuel cladding. The new materials should have stronger properties than zirconium alloys, which can provide sufficient fault-tolerant ability in accidents [2,3,4,5,6,7].

SiC ceramics are considered as potential materials for nuclear-fuel cladding because of their excellent mechanical and irradiation properties [6,7,8]. However, it is difficult to join SiC ceramics, especially with nuclear-fuel cladding requiring strict accuracy using traditional fusion welding, due to their high melting temperature (~2973 K). Compared with fusion welding, the brazing technique introduces little thermal deformation of parent materials, meeting the accuracy requirement in nuclear engineering. Meanwhile, the bonding temperature used in brazing is usually lower than that in fusion welding, making the manufacturing process simple and more easily controlled. Considering this, the brazing technique is considered to be the most promising method to join SiC ceramics in nuclear fields [9]. Many filler alloys, such as Cu-based, AgCu-based, Ni-based, and Co-based alloys, were developed to join SiC ceramics. However, most of them cannot be used at the service temperatures of nuclear-fuel cladding (923–1023 K). High-temperature fillers were merely reported [10,11,12,13,14]. Shujie Li et al. [12] used Ni–Cr–SiC powders to join SiC ceramics at 1673 K. The Ni–Ti filler was reported to join SiC ceramics at temperatures up to 1823 K [15]. It is well known that the matrix will be significantly degraded when using such a high brazing temperature. Xiong et al. [13] added Si and B as melting-point depressants into Co–Fe–Ni–Cr–Ti alloy to achieve good high-temperature strength with a relatively low bonding temperature. The three-point bending strength of assemblies brazed at 1423 K for 10 min was 142.2 MPa, 162.3 MPa, 188.2 MPa, and 181.5 MPa, when testing at room temperature, 973 K, 1073 K, and 1173 K, respectively. To develop high-temperature filler alloys, the rational design of chemical composition in the filler alloy is indispensable. Considering this, the current work focuses on developing a new high-temperature filler alloy for the joining of SiC ceramics.

In this work, a new high-temperature filler alloy containing Au, Ni, Pd, and Ti was designed to achieve the high-quality bonding of SiC ceramics. Au is the primary element in the filler alloy, because its superior plasticity effectively alleviates the residual stress caused by thermal mismatch between the filler and matrix. It can be seen from the Au–Ni phase diagram [16] that Au and Ni can be infinitely miscible at any proportion, and they transform to liquid at a relatively low temperature (1223 K), when the content of Ni is about 18 wt.% in Au–Ni. Therefore, Au_82_Ni_18_ (wt.%) was determined to be the matrix of the filler alloy. Incorporation of 4 wt.% Pd into Au–Ni improves its oxidation resistance. To enhance the wettability of filler alloy to SiC, 4 wt.% Ti was also incorporated into the Au–Ni alloy because of its reactivity with SiC. Based on the above analysis, the filler alloy with the composition of (Au_79_Ni_17_Pd_4_)_96_Ti_4_ (wt.%) was designed to join the SiC ceramics. It can be found from Au–Ni–Pd ternary phase diagram [17] that the melting point of Au_79_Ni_17_Pd_4_ (wt.%) is approximately 1423 K. Considering this, the experiments for the joining of SiC ceramics were carried out at 1423–1573 K for 10–90 min. The typical microstructure in the joints was analyzed and the formation mechanism of joints was discussed in detail. Meanwhile, the effects of brazing temperature and holding time on microstructure and fracture morphology of the joints were studied.

## 2. Materials and Methods

In this work, the SiC ceramics used had a purity of 97 wt.% and contained no free Si. They were cut into two sizes of 10 mm × 8 mm × 4 mm and 4 mm × 4 mm × 4 mm (bonding surface: 4 mm × 4 mm). The filler alloy was composed of Au, Ni, Pd, and Ti foils, which were purchased from Alfa Aesar Chemicals Co., Ltd (Shanghai, China). The foils with a purity over 99.5 wt.% had a thickness of 0.025 mm. The SiC ceramics were ground and polished prior to brazing. The filler foils were prepared through the following steps: firstly, the Au foils were cut into a size of 4 mm × 4 mm × 4 mm, and then weighed by an electronic analytical balance with an accuracy of 0.1 mg. Secondly, the needed weights of Ni, Pd, and Ti foils were calculated and prepared according to the designed chemical composition.

All materials were ultrasonically cleaned in acetone for 30 min and dried prior to assembly. After that, the foils were sandwiched between SiC ceramics and fixed with the cyanoacrylate glue that would be completely evaporated at 393 K. During brazing, a pressure of ~0.01 MPa was imposed on the assembly by placing a molybdenum block on top of the assembly. The melting temperature of Mo is 2896 K, which maintains stability at the brazing temperatures. During brazing, the assemblies were first heated to 573 K at a rate of 10 K/min, and then held for 30 min to evaporate the glue. The assemblies were then elevated to the brazing temperatures (1423–1573 K) at a rate of 10 K/min, isothermally soaked for 10–90 min, and finally cooled to the room temperature at a rate of 5 K/min. The vacuum in the furnace should be maintained at ~10^−3^ Pa during the whole process, which would protect the joints from oxidation and reaction with impurities in the atmosphere. The obtained joints were mounted, sectioned, and polished with a standard process. The microstructure of joints was examined using scanning electron microscopy (SEM, FEI Quanta200F, Hillsboro, OR, USA) coupled with energy-dispersive X-ray spectroscopy (EDS, Bruker Nano Xflash Detector 5010, Fitchburg, WI, USA). The shear strengths of joints were tested, and the fracture morphologies were observed by SEM.

## 3. Results

### 3.1. Typical Microstructure of the SiC/SiC Joint

**Figure 1:**
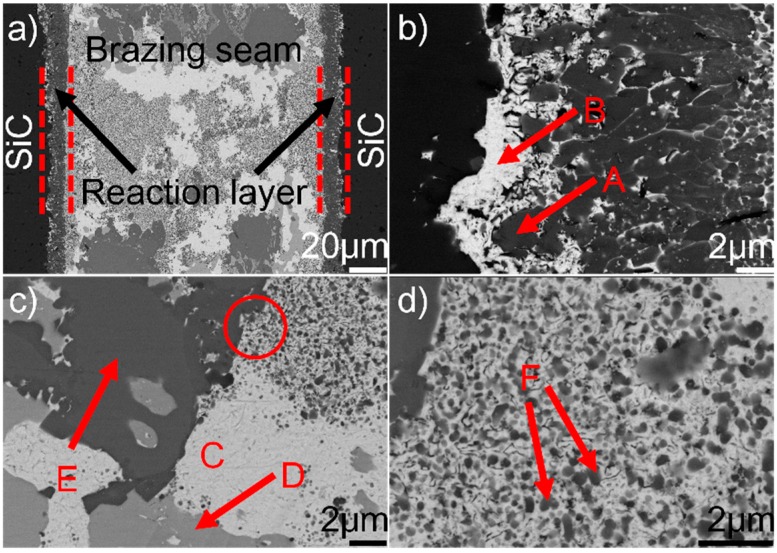
(**a**) Back-scattered electron (BSE) micrographs of the typical microstructure in the SiC/(Au_79_Ni_17_Pd_4_)_96_Ti_4_/SiC joint brazed at 1523 K for 10 min; (**b**) magnified morphology of the SiC/braze interface; (**c**) magnified morphology of the typical braze zone; (**d**) magnified morphology of red cycle in (**c**).

Figure 1 shows the typical microstructure of SiC/SiC joint brazed using (Au_79_Ni_17_Pd_4_)_96_Ti_4_ (wt.%) at 1523 K for 10 min. It can be seen from Figure 1a that a good wetting and intimate contact were achieved at the substrate/filler alloy interface. No obvious defects (pores and cracks) can be inspected. A continuous and compact reaction layer (Figure 1a) can be seen at the SiC/braze interface. The reaction layer, as revealed in Figure 1b, contains two phases, black phase A and white phase B, with black phase predominance. The braze seam (Figure 1c) is primarily made up of three phases: white phase C, light-gray phase D, and dark-gray phase E. Some nano-sized particles (F), as indicated in Figure 1d, are distributed in phase C.

**Figure 2:**
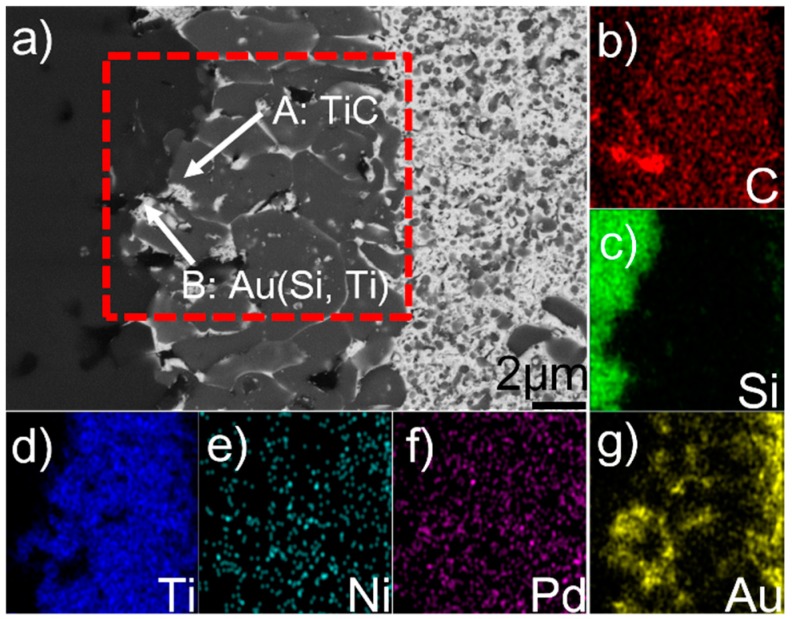
(**a**) BSE micrograph showing the detailed microstructure of the SiC/braze interface, and relevant elemental distribution maps of the red rectangle in (**a**): (**b**) C; (**c**) Si; (**d**) Ti; (**e**) Ni; (**f**) Pd; (**g**) Au.

Figure 2 displays the microstructure of the reaction layer at the SiC/braze interface and elemental map distribution. Table 1 lists the EDS results of phases in the joint. The reaction layer, as mentioned, contained two phases (A and B). Phase A, as can be seen from Figure 2b,d, is mainly enriched with C and Ti. The C/Ti atomic ratio is close to 1, suggesting that this phase contains TiC. The TiC was formed through the reaction between Ti and SiC, which was reported in References [18,19,20]. Phase B, as indicated in Figure 2g, is enriched with Au. Due to the fact that it has Au (51.1 at. %), Si (32.5 at. %), Ti (13.0 at. %), and Ni (3.5 at. %), it can be inferred that phase B is actually an Si- and Ti-enriched Au-based solid solution, namely Au (Si, Ti). The SiC ceramic was well wetted by the Au (Si, Ti) and TiC, forming a strong bonding interface. In general, pure Au cannot wet SiC [21]. However, it should be noted that 32.5 at. % Si was dissolved in Au (Si, Ti). The equilibrium wetting angle (θ) of pure Au/SiC is 150–110° at 1336–1703 K, while a strong wettability of Au + Si was found on the SiC (θ < 20°) [22]. Therefore, the Au (Si, Ti) could wet the SiC in this work, because of silicon chemisorption at the braze/SiC interface with the formation of strong covalent-like bonds between Si and SiC [23]. Based on the above analysis, the reaction layer at the SiC/braze interface is mainly composed of TiC, in which some Au (Si, Ti) is distributed.

**Figure 3:**
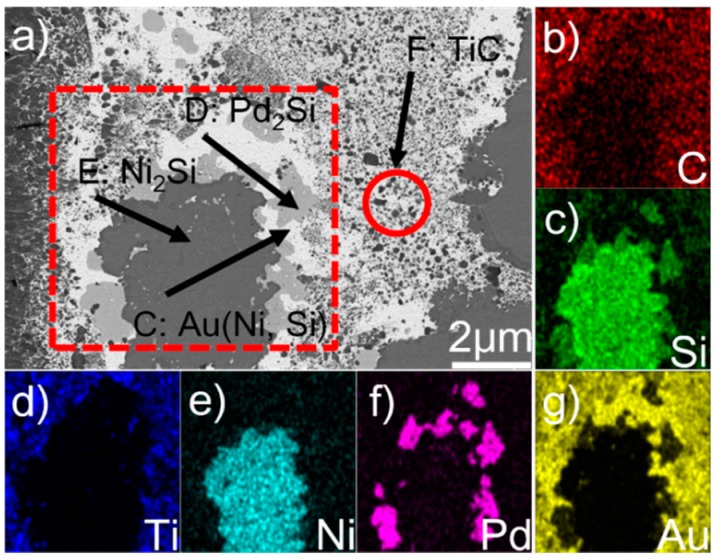
(**a**) BSE micrograph showing the detailed microstructure in the braze seam, and relevant elemental distribution of the red rectangle in (**a**): (**b**) C; (**c**) Si; (**d**) Ti; (**e**) Ni; (**f**) Pd; (**g**) Au.

Figure 3 displays the microstructure of the braze seam and elemental map distribution. The element Au, as can be seen from Figure 3a,g, is aggregated in phase C. The EDS results show that it has Au (95.0 at.%), Ni (3.1 at.%), Si (1.0 at.%), and Ti (0.6 at.%) (see Table 1). It can be found from Au–Ni and Au–Si diagrams that there is no compound between Au and Ni or Au and Si with the atomic ratio obtained from the EDS results. In addition, the solubility of Ni in Au was infinitely soluble at room temperature, while the solubility of Si in Au was less than 10 at.%. It can be then deduced that phase C actually involves Au-based solid solutions, namely Au (Ni, Si). Phase D is concentrated with Pd and Si (Figure 3c,f) and the Pd/Si atomic ratio is close to 2 (Table 1). This phase was then determined to be Pd_2_Si. Ni and Si are the primary elements in phase E (see Figure 3c,e). According to the Ni/Si atomic ratio (~2), this phase was identified to be Ni_2_Si. Phase F was identified to be TiC, since it basically consisted of C and Ti (Table 1).

**Figure 4:**
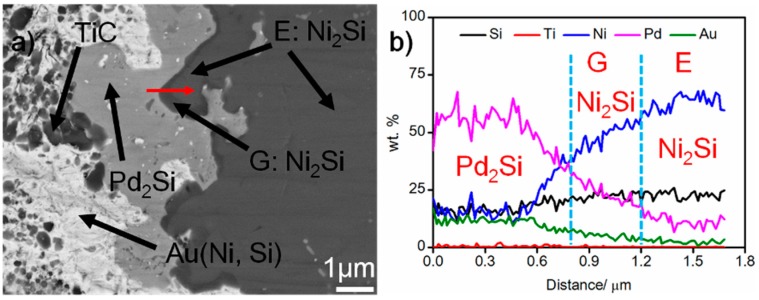
(**a**) SEM morphology of the braze seam, and (**b**) elemental distribution across the red scanning line in (**a**).

Figure 4 shows the SEM morphology of the braze seam. It can be seen from the figure that some black phases (labeled G) take place at the Pd_2_Si/Ni_2_Si interfaces. The elemental line scanning results indicate that the content of Pd in G is higher than that in E, while the content of Si and Ni decreases (see Figure 4b). Considering this, phase G should also be Ni_2_Si. The composition divergence between phase E and G can also be demonstrated in Table 1. Based on this, the braze seam is mainly made up of Au (Ni, Si), Pd_2_Si, and Ni_2_Si. Moreover, a great amount of nano-sized TiC particles are dispersed in the Au (Ni, Si) layer.

Based on the above microstructural analysis, the formation mechanism of the joint can be discussed; the filler foils deformed and came into contact with each other closely with increasing temperature. When the heating temperature reached 1223 K, the Au and Ni foils melted, and Pd and Ti were subsequently dissolved into the liquid. As the heating temperature was elevated to 1423 K, all the foils melted. The braze seam was actually a multi-component alloying system.

At the SiC/filler alloy interface, when Au–Ni was transformed into liquid, the covalency of SiC crystals weakened because some Ni atoms could infiltrate into their surfaces, leading to the decomposition of SiC below its intrinsic decomposition temperature [24]. The Si atoms released entered into the liquid and improved the wettability of Au toward the SiC. In addition, dissolved Ti was also concentrated at the SiC/braze interface due to its affinity, and reacted with the SiC, leading to the production of a thin TiC layer. Production of TiC promoted the spreading of molten filler alloy on the SiC. After that, the liquid filler alloy wetted the substrates and spread on them. With the interfacial reaction continuing, the TiC reaction layer became more compact and thicker. After that, the growth rate of TiC interfacial layers was mainly controlled through a diffusion mechanism. In other words, Ti needed to migrate the formed TiC to react with the SiC. It should be mentioned that the reaction layer at the SiC/braze interface was made up of TiC and Au (Si, Ti). It can be then inferred that the wettability of SiC by the braze was controlled through two wetting mechanisms: non-reactive wetting (Au (Si, Ti)) and reactive wetting (TiC).

In the braze seam, under the effect of concentration gradient, the C and Si atoms decomposed at the SiC/braze interface diffuse toward the braze seam. At the temperature range of 1223–1523 K, the Gibbs free energies of formation for TiC, Pd_2_Si, and Ni_2_Si are about −175 kJ/mol, −20 kJ/mol, and −135 kJ/mol, respectively [25,26,27]. All energies are negative, indicating that these compounds could be spontaneously generated. The C atoms reacted with Ti and generated nano-sized TiC particles in the braze seam. Considering the fact that the melting points of Pd_2_Si and Ni_2_Si are 1604 K and 1565 K, respectively [27,28], Pd_2_Si would be formed first, followed by Ni_2_Si. In cooling, the Au (Ni, Si) layer was solidified when the temperature was reduced to about 1337 K.

### 3.2. Effect of Brazing Temperature on the Microstructure of the Joints

**Figure 5:**
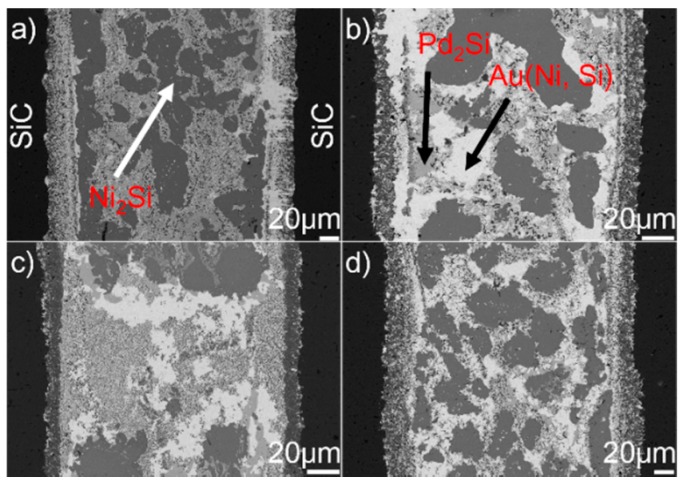
BSE images showing the microstructure of the SiC/SiC joints brazed for 10 min at different temperatures: (**a**) 1423 K; (**b**) 1473 K; (**c**) 1523 K; (**d**) 1573 K.

**Figure 6:**
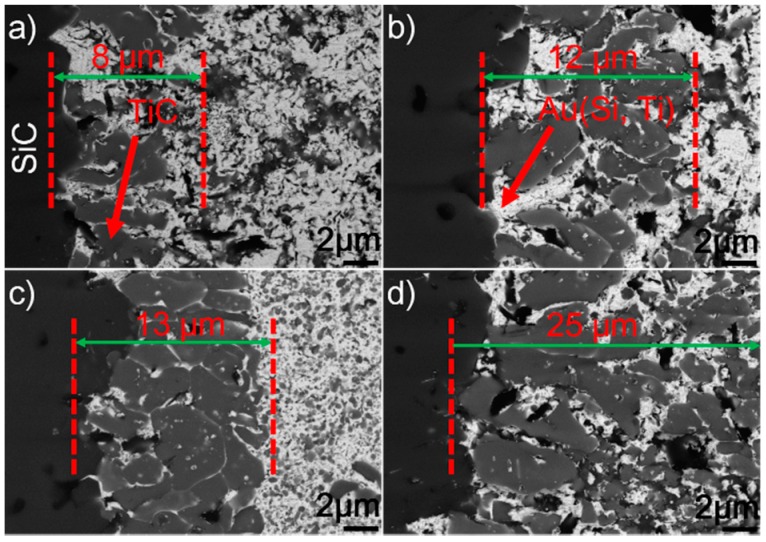
BSE images showing the reaction layer at the SiC/braze interface obtained for 10 min at (**a**) 1423 K, (**b**) 1473 K, (**c**) 1523 K, and (**d**) 1573 K.

Figure 5 presents the SEM morphologies of the SiC/SiC joints obtained at 1423–1573 K for 10 min. It can be seen from the figure that an interfacial reaction layer can always be observed at different joining conditions. No obvious defects can be inspected. Figure 6 displays the detailed microstructure at the SiC/braze interfaces. It can be shown from the figure that the interfacial reaction layer always consists of TiC and Au (Si, Ti). Upon elevating the brazing temperature, its thickness increases. When the reaction layer was first formed, its growth was then controlled through a diffusion mechanism [29]. It is noteworthy that the diffusion rate was related to concentration gradient and temperature. According to the theory of mass transfer, the diffusion process was driven by the concentration gradient. Furthermore, Fick’s first law states that the diffusion flux is proportional to the concentration gradient [30]. In this work, the concentration of Ti was equal in each joining condition. Therefore, increasing the brazing temperature would increase the atomic motion and, thus, the diffusion rate. The element Ti passing through the already-formed TiC reaction layer was accelerated at a higher brazing temperature, speeding up the interfacial reaction. An increase in brazing temperature produced a thicker reaction layer at a fixed soaking time.

**Figure 7:**
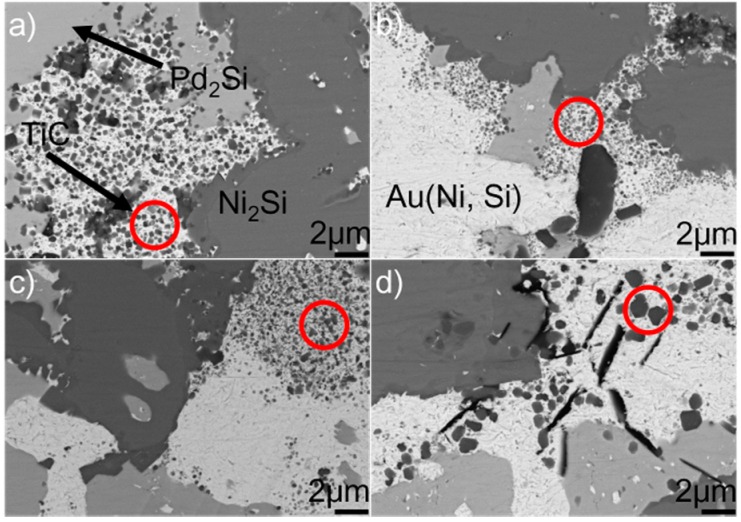
BSE images showing the braze seam obtained for 10 min at (**a**) 1423 K, (**b**) 1473 K, (**c**) 1523 K, and (**d**) 1573 K.

Figure 7 shows the typical microstructure in the braze seam obtained at 1423–1573 K for 10 min. It can be seen from the figure that the constitution of braze seam is basically identical at different joining conditions. Pd_2_Si and Ni_2_Si within the braze seam show no remarkable change, while the TiC particles within the Au (Ni, Si) layer are coarsened at a higher brazing temperature. Some black needles can be observed in Figure 7d, and they were identified to be the carbon. These carbon black needles were brought into Au-based solid solutions via diamond discs during sample preparation. They could not be observed in Pd_2_Si and Ni_2_Si because they had a higher hardness than the Au-based solid solutions. The filler alloy, as mentioned, melted completely at 1423 K, and formation of TiC, Pd_2_Si, and Ni_2_Si within the braze seam then took place. Therefore, the growth of Pd_2_Si and Ni_2_Si was actually limited by the concentrations of Pd and Si, respectively. The nucleation and coarsening of Pd_2_Si ended when the element Pd was exhausted. The Si atoms decomposed from the SiC ceramics reacted with Ni to produce Ni_2_Si within the braze seam. It can be, therefore, inferred that the rate-limiting process for the Ni_2_Si production should be the diffusion of Si, which was hindered by the TiC reaction layer at the SiC/braze interface. After the Si was depleted in the braze seam, Ni_2_Si could not grow. Compared to Si, the C atoms decomposed from the SiC could diffuse into the braze seam easily due to their small atomic size. Upon increasing the brazing temperature, more C atoms diffused toward the braze seam, leading to the growth of TiC particles within the Au (Ni, Si) layer.

### 3.3. Effect of Soaking Time on the Microstructure of the Joints

**Figure 8:**
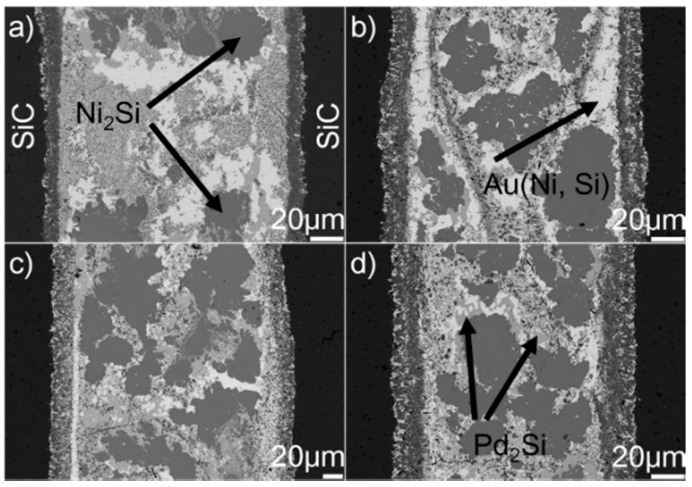
BSE images indicating the microstructure of the SiC/SiC joints brazed at 1523 K for different soaking times: (**a**) 10 min; (**b**) 30 min; (**c**) 60 min; (**d**) 90 min.

**Figure 9:**
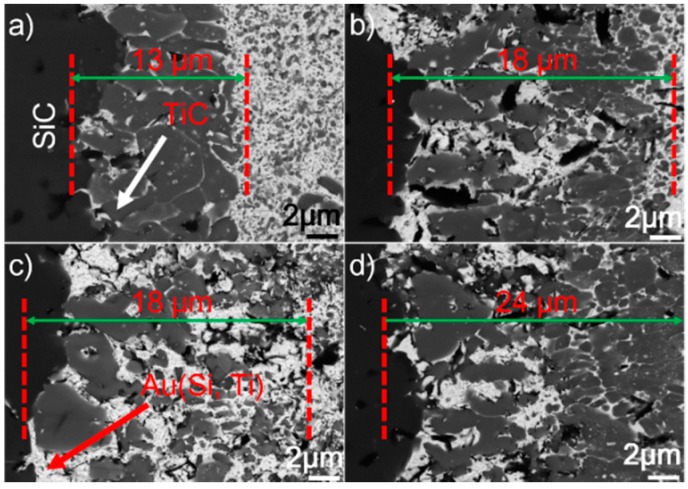
BSE images indicating the reaction layer at the SiC/braze interface obtained at 1523 K for different soaking times: (**a**) 10 min; (**b**) 30 min; (**c**) 60 min; (**d**) 90 min.

Figure 8 displays the microstructure of the SiC/SiC joints brazed at 1523 K for 10–90 min. It can be seen from the figure that an obvious interfacial reaction layer can always be observed in all joints. Figure 9 shows the detailed microstructure of the interfacial reaction layer at the SiC/braze interface. The reaction layer, as revealed, became thicker, and the TiC particles within the reaction layer grew larger for a prolonged soaking time. Controlled by the diffusion mechanism, the interfacial reaction at the SiC/braze interface, as mentioned, started during the heating stage. An extension of the soaking time increased the interfacial reaction time, leading to the growth of the reaction layer.

**Figure 10:**
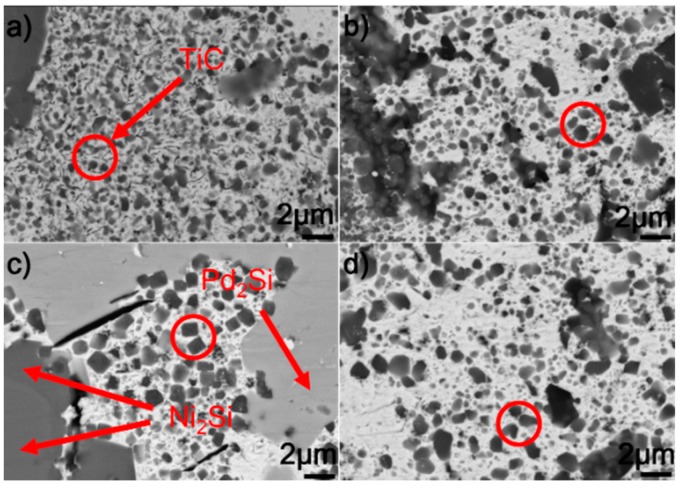
BSE images indicating the braze seams obtained at 1523 K for different soaking times: (**a**) 10 min; (**b**) 30 min; (**c**) 60 min; (**d**) 90 min.

Figure 10 shows the SEM morphologies of the brazing seam obtained at 1523 K for 10–90 min. Upon prolonging the soaking time, the TiC particles within the Au (Ni, Si) layer grew gradually. When the soaking time was 10 min, the average diameter of TiC particles was generally less than 0.5 μm. Upon extending the soaking time to 90 min, most of the TiC particles had a particle size of 1.0 μm. Prolonging the extension time provided more time for the TiC particles within the Au (Ni, Si) layer to grow, contributing to their coarsening. The morphologies of Pd_2_Si and Ni_2_Si obtained for different soaking times, as shown in Figure 8, had no significant variation. The nucleation and growth of Pd_2_Si and Ni_2_Si, as mentioned, were limited by the contents of Pd and Si, respectively. After they were depleted, the growth of Pd_2_Si and Ni_2_Si accordingly ended.

### 3.4. Fracture Morphologies of the Joints

**Figure 11:**
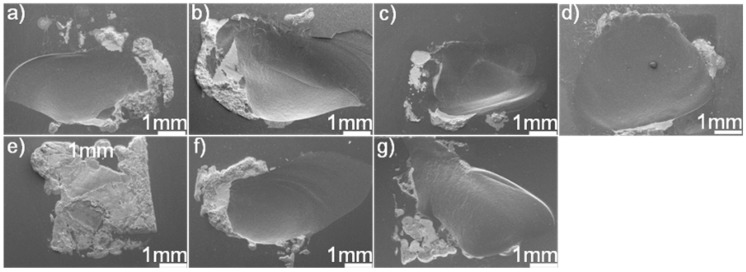
SEM fracture morphologies of the SiC/SiC joints brazed using (Au_79_Ni_17_Pd_4_)_96_Ti_4_ (wt.%) under different joining conditions: (**a**) 1423 K/10 min; (**b**) 1473 K/10 min; (**c**) 1523 K/10 min; (**d**) 1573 K/10 min; (**e**) 1523 K/30 min; (**f**) 1523 K/60 min; (**g**) 1523 K/90 min.

Figure 11 displays the fracture morphologies of shear specimens. It can be seen from the figure that most of the joints fractured in the SiC ceramics adjacent to the braze seam. The fracture surface suggested that the filler alloy was well bonded to SiC ceramics. A large residual stress residing in the joints caused the fracture. Upon cooling, the deformation of the braze zone and substrate was discordant, resulting in a tensile residual stress in the ceramics adjacent to the braze seam. The bowed fracture surfaces suggested that the tensile residual stress should be the primary cause for the joint fracture. In general, the residual stress was proportional to thermal mismatch between the filler alloy and substrate. Table 2 lists the coefficient of thermal expansion (CTE) and Young’s modulus (E) for some phases within the SiC ceramic joint [31,32,33,34,35,36]. The CTE (14.2 × 10^−6^ K^−1^) of Au is far larger than that of the SiC (3.8 × 10^−6^ K^−1^), while the CTEs of TiC, Pd_2_Si, and Ni_2_Si are smaller than that of the Au. Incorporation of TiC, Pd_2_Si, and Ni_2_Si with a relatively lower CTE into the braze would decrease the CTE of the braze [37,38,39], leading to the decrease of CTE mismatch between the SiC and filler alloy. This effect would be beneficial to lower the stress level in the joint. However, it should be noted that the filler alloy with a certain ability of plastic deformation was also important to release the residual stresses during joining. Increasing the volume fraction of hard phases into the braze would decrease the CTE mismatch but also deteriorate its plastic deformation. Considering this, a balance should be reached between them. The stress level in the joint, as well known, is closely related to the microstructure. In this work, the brazing temperature and soaking time had a relatively pronounced effect on the thickness of the reaction layer at the SiC/braze interface, while it showed negligible influence on the volume and amount of Pd_2_Si and Ni_2_Si within the braze.

In this work, the phases within the joint were identified and their formation mechanism was clarified. In addition, the effect of brazing temperature or soaking time on the microstructure of the joints was investigated. The above facts suggested that the designed high-temperature filler alloy has considerable potential in joining SiC ceramics in nuclear-fuel cladding. We also tested the shear strength of joints with different brazing parameters, and the results showed that the shear strength was around 100 MPa. A large stress level in the brazed joint led to the shear strength obtained, which should be ascribed to the appearance of lots of hard phases (such as TiC with a Young’s modulus of 460 GPa) having a poor plastic deformation in the braze seam. The mechanical properties of the SiC/SiC joints are expected to be improved through optimizing the brazing parameters and chemical compositions of the filler alloy, which will be investigated in the near future.

## 4. Conclusions

(1) The typical microstructure of SiC/SiC joint brazed using (Au_79_Ni_17_Pd_4_)_96_Ti_4_ (wt.%) can be described as SiC/reaction layer/braze/reaction layer/SiC. The reaction layer at the SiC/braze interface was mainly composed of the TiC and Au (Si, Ti) layers, which meant that the SiC ceramics were wetted through a mixed mechanism of non-reactive wetting (Au (Si, Ti)) and reactive wetting (TiC). Dissolution of Si into the Au (Ni, Si) layer was a critical factor to promote wettability toward the SiC ceramics.
(2) The braze in the joint was mostly constituted by Ni_2_Si, Pd_2_Si, and Au (Ni, Si). Apart from that, a substantial amount of nano-sized TiC particles were dispersed within the Au (Ni, Si) layer. In the heating stage, TiC was first generated in the braze, followed by Pd_2_Si and Ni_2_Si. The Au (Ni, Si) layer was solidified in cooling.
(3) For the brazing parameters selected, increasing the brazing temperature or extending the soaking time promoted the growth of the reaction layer at the SiC/braze interface and coarsening of TiC particles within the Au (Ni, Si) layer. However, different brazing parameters used caused a negligible effect on the morphologies of Pd_2_Si and Ni_2_Si within the braze, because the rate-limiting factor controlling the nucleation and growth of the Pd_2_Si and Ni_2_Si was the concentrations of Pd and Si within the braze, respectively.
(4) The SiC/SiC brazed joints fractured on the SiC, suggesting that the filler alloy was well bonded with the SiC. The residual thermal stresses should be the critical factor inducing the joint failure. To reduce the stress level, the joint microstructure should be modified through optimizing the brazing parameters coupled with chemical composition of the filler alloy.

## Figures and Tables

**Table 1:** Energy-dispersive X-ray spectroscopy (EDS) results of phases in the SiC/SiC joint.

	C	Si	Ti	Ni	Pd	Au	Phases
A	48.3	0.4	50.8	-	-	0.5	TiC
B	-	32.5	13.0	3.5	-	51.1	Au (Si, Ti)
C	-	1.0	0.6	3.1	-	95.0	Au (Ni, Si)
D	-	32.0	0.2	8.0	55.1	4.8	Pd_2_Si
E	-	37.8	0.2	60.8	1.1	-	Ni_2_Si
F	56.24	3.79	27.44	-	-	12.53	TiC
G	-	43.4	1.3	49.5	4.6	1.3	Ni_2_Si

**Table 2:** Coefficients of thermal expansion (CTEs) and Young’s moduli € of related phases in the joint [31,32,33,34,35,36].

	Au	SiC	TiC	Pd_2_Si	Ni_2_Si
CTE (×10^−6^ K^−1^)	14.2	3.8	7.4	13.2	9.2
E (GPa)	59	450	460	86	190
